# Barcode extension for analysis and reconstruction of structures

**DOI:** 10.1038/ncomms14698

**Published:** 2017-03-13

**Authors:** Cameron Myhrvold, Michael Baym, Nikita Hanikel, Luvena L Ong, Jonathan S Gootenberg, Peng Yin

**Affiliations:** 1 Wyss Institute for Biologically Inspired Engineering, Harvard University, Boston, Massachusetts 02115, USA; 2 Department of Systems Biology, Harvard Medical School, Boston, Massachusetts 02115, USA

## Abstract

Collections of DNA sequences can be rationally designed to self-assemble into predictable three-dimensional structures. The geometric and functional diversity of DNA nanostructures created to date has been enhanced by improvements in DNA synthesis and computational design. However, existing methods for structure characterization typically image the final product or laboriously determine the presence of individual, labelled strands using gel electrophoresis. Here we introduce a new method of structure characterization that uses barcode extension and next-generation DNA sequencing to quantitatively measure the incorporation of every strand into a DNA nanostructure. By quantifying the relative abundances of distinct DNA species in product and monomer bands, we can study the influence of geometry and sequence on assembly. We have tested our method using 2D and 3D DNA brick and DNA origami structures. Our method is general and should be extensible to a wide variety of DNA nanostructures.

## Abstract (web summary)

Techniques for structural characterization and quantification of DNA origami are still poorly developed, despite advances in other aspects of DNA nanotechnology. Here, the authors combine barcoding and next generation sequencing to simultaneously image and quantify self-assembled DNA nanostructures.

The discovery in 1982 that DNA can self-assemble into designed structures initiated the field of structural DNA nanotechnology^1^. Over the past few decades, the field of structural DNA nanotechnology has produced a stunning array of two- (2D) and three-dimensional (3D) structures^2^,^3^,^4^,^5^,^6^,^7^,^8^,^9^,^10^,^11^,^12^,^13^. These structures have been used for a variety of applications, such as protein structure determination^14^, enzyme scaffolding^15^,^16^,^17^, photonics^18^,^19^,^20^ and drug delivery^21^,^22^,^23^. The standard workflow is typically as follows: structures are designed on a computer, component oligonucleotides are ordered and synthesized commercially, the structures are assembled in the lab, and then the structures are characterized using imaging or other analytical methods, including gel electrophoresis. This design-build-test process can be iterated several times if necessary to achieve a design with high performance.

Most aspects of the design-build-test cycle have been markedly improved over the past few decades. Structure design is much easier now than it was in 1982, as evidenced by new design paradigms (DNA origami^5^, DNA bricks^8^, gridiron^6^ and 3D polyhedral meshes^10^) and software packages for automating structure design and analysis (NUPACK^24^, caDNAno^25^ and CanDo^26^). The synthesis of the oligonucleotides that form the components of a structure is growing exponentially cheaper. Structure assembly is also faster and easier than ever before, with the recent demonstration of isothermal assembly protocols for DNA origami and DNA brick structures^27^,^28^. As a result of these combined advances, one can now design and assemble multiple structure designs in a single round of testing.

Despite the many advances in structure design, synthesis and assembly, structure imaging remains low throughput and requires considerable time and effort per structure. In recent years, new technologies such as fast-scan atomic force microscopy (fast-AFM) and cryo-electron microscopy^29^,^30^ have increased the speed and resolution with which DNA nanostructures can be imaged, but still require substantial equipment investments and expertise to use to their fullest extent. Super-resolution optical microscopy techniques such as DNA-PAINT^31^ have proven very helpful for imaging structures with multiple orthogonal labels^32^ in 3D^33^, but they require labelling structures with organic dyes or single-stranded extensions. In spite of these advances, it remains difficult to characterize the component composition of multidimensional DNA nanostructures in a high-throughput, label-free manner.

In addition to imaging methods, several methods based on gel electrophoresis can be used to analyse DNA structure assembly. These methods compare the amount of material present in monomer, product and aggregate bands, and measure structure-wide average quality or the site-specific incorporation of labelled oligonucleotides. The simplest such label is a fluorescent intercalating dye (for example, Sybr Safe), but de-Bruijn probes can provide more quantitative estimates of the average structure quality^34^. In some cases, one cares more about the local assembly of particular structural features, rather than overall structure quality. In these cases, fluorescently labelled oligonucleotides are typically employed to measure site-specific incorporation^35^, or fluorescence resonance energy transfer is used to measure the co-localization of two structure components^17^,^36^. These methods are generally simpler to employ than imaging, but they do not provide detailed information about the component composition of a structure with single component strand resolution. Thus, measuring the incorporation efficiency of all of the components of a DNA structure remains challenging.

A candidate method that could provide detailed quantitative information about the component composition of DNA nanostructures is next-generation DNA sequencing (NGS). Since each component strand in a fully addressable DNA nanostructure has a unique DNA sequence, it should be possible to obtain information about the composition of an entire structure with single brick or staple resolution. NGS thus has higher multiplexing capabilities than site labelling methods, which typically are limited to labelling a few component oligonucleotides at a time. Unlike imaging methods, NGS allows for many samples to be processed in parallel using sequence barcodes, thereby increasing the throughput of the method substantially compared with imaging methods. Also, since the sequencing data are collected as an unbiased class average of many individual structures, they provide a rich picture of the statistics of self-assembly. Furthermore, the cost of NGS has dropped exponentially over the past few years, making this an increasingly attractive and affordable analysis technique. NGS has been used by biologists to measure RNA expression levels^37^, ribosome activity^38^, transcription elongation^39^ and protein–DNA interactions^40^, thus it should be possible to apply the method to study the self-assembly of DNA nanostructures in a quantitative fashion.

Here we introduce a method for studying the assembly of DNA nanostructures that uses NGS to quantify the relative incorporation of staples or bricks. The method works by assembling structures, segregating and isolating products, and unincorporated strands using gel electrophoresis and ligating barcoded adaptor sequences to the ends of denatured staples or bricks. Once the ligations are complete, the resulting libraries can be purified, amplified and analysed using NGS. We call our method barcode extension for analysis and reconstruction of structures, or BEARS. Using BEARS, we demonstrate the simultaneous quantification of each of the components of 2D and 3D DNA brick and DNA origami nanostructures. Thus, BEARS can be used to study the assembly of a wide range of self-assembled DNA nanostructures.

## Results

### Developing a barcode ligation strategy

**Figure 1:**
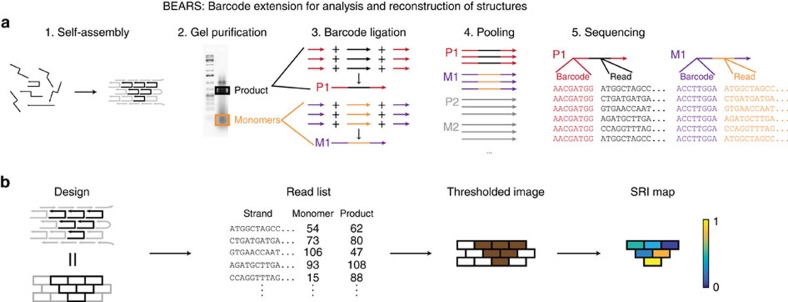
BEARS uses sequencing to assay DNA nanostructure assembly. We show schematics of each of the five major parts of BEARS: structure assembly, purification, barcode ligation, pooling and next-generation sequencing (**a**). Structures are assembled, and well-assembled products and unincorporated monomers are isolated on an agarose gel. Barcodes and sequencing adaptors are ligated onto each sample in separate reactions, and then samples are pooled and sequenced using NGS. We also show an overview of the data analysis pipeline used to generate a quantitative, spatial map of strand incorporation (**b**). We start with a molecular canvas and a structure design containing a subset of the DNA brick strands within the canvas. After self-assembly and sequencing, we have a list of read counts for each brick strand in the product and monomer bands. We threshold the sequencing data to determine which brick strands are part of the assembled structure and which are not (see the Methods section for details). Finally, we compute the SRI for each strand that passed the threshold (see the Methods section for details).

The BEARS protocol is divided into five basic parts as follows: structure assembly, product and monomer purification, denaturation and barcode ligation, sample pooling, and NGS (Fig. 1a). Structure assembly, product purification and NGS involve standard procedures, but the barcode ligation step presents a unique challenge (Supplementary Fig. 1). Ideally, one would like to attach barcodes in an efficient, unbiased and sequence-independent manner. To do this, we developed a barcode ligation strategy that uses single-stranded DNA (ssDNA) ligation. First, a 5′-phosphorylated adaptor with a 3′-dideoxycytosine modification is ligated to the 3′-end of a brick or staple using T4 RNA ligase 1. The ligation product is then purified on a PAGE gel (Supplementary Fig. 2), phosphorylated and a barcoded adaptor is added to the 5′-phosphate using T4 RNA ligase 1. This doubly ligated product is column-purified and amplified using PCR. The resulting samples (isolated from the product band, the monomer band or the input oligonucleotide mix for each structure design) are pooled to enhance the throughput of the method, and then sequenced using NGS. Although BEARS requires structure purification, this is not a limitation because structures are denatured immediately after purification. Thus, we do not expect purification to alter the component composition of the structures analysed using BEARS.

Our method differs from a previously published method for sequencing ancient ssDNA^41^, in which we use a different enzyme for ssDNA ligation, perform size-selection using PAGE and do not use primer extension. Furthermore, it should be noted that methods such as ribosome profiling^38^ use ssRNA ligation to attach adaptor sequences and are therefore not suitable for sequencing components of DNA nanostructures. For further details regarding sequencing library preparation, see the Methods section and Supplementary Figs 1 and 2.

### Sequencing data analysis

NGS sequencing data files are scanned to look for reads corresponding to strands present in a DNA origami design or DNA brick canvas (Supplementary Fig. 1). This yields a list of read counts for each strand (for a histogram of the read counts, see Supplementary Fig. 3). Read fractions are computed from the read count lists by dividing the read count for each strand by the sum of all of the read counts in a sample. Next, we determine which strands are present in the structure by taking the ratio of the read fraction in the product band to the read fraction in the entire canvas or origami design. This yields either a unimodal or bimodal lognormal distribution, with the rightmost peak corresponding to well-incorporated strands and the left-most peak corresponding to strands that were not present in the structure (Supplementary Fig. 4). If one peak is present, then all strands are well-incorporated, which is typically the case with DNA origami structures. When necessary, we apply a threshold to separate these two groups of strands. Further analysis is performed on the strands that passed the threshold, as described below.

**Figure 2:**
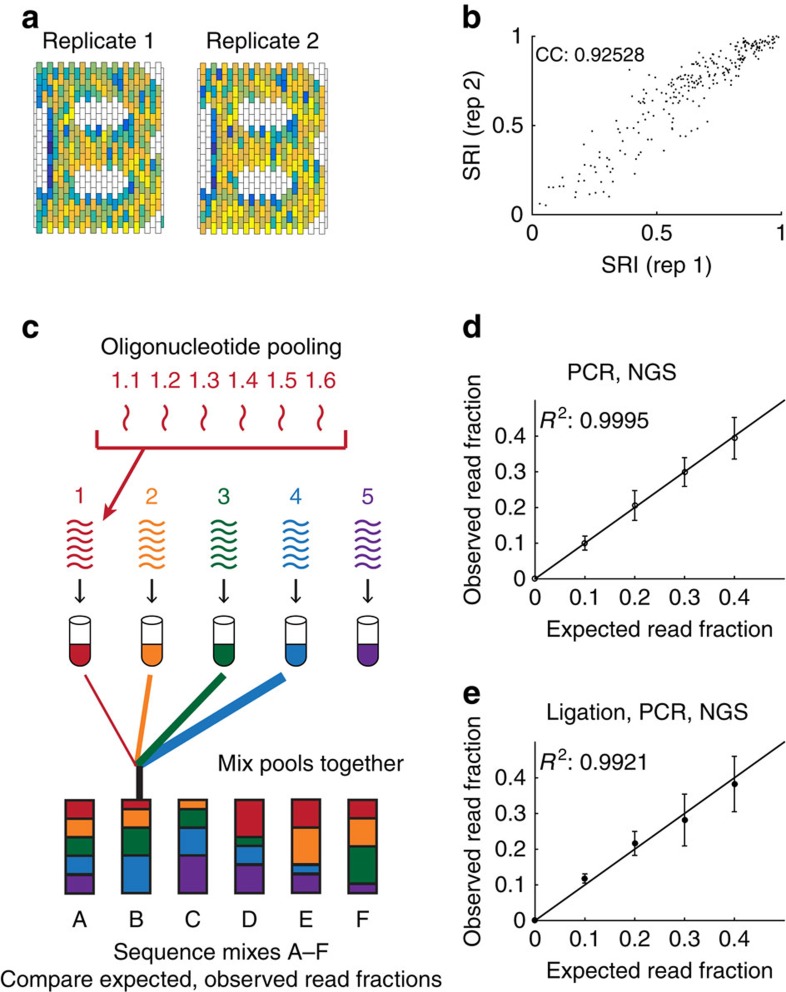
Sequencing is reproducible and quantitative. We assembled and sequenced a 2D DNA brick structure (the letter B) on two separate occasions using BEARS, and calculated the SRI for each replicate experiment (**a**). A scatterplot of the SRI values from each replicate is shown (**b**). The correlation coefficient between the two replicates is indicated in the upper left corner of the graph. We performed oligo mixing experiments in which five pools containing six unique oligonucleotides species each were mixed together with varying stoichiometries (**c**). We compared the observed and measured read fractions of these oligonucleotide pools after PCR and NGS using pre-extended oligonucleotides (**d**) and after ligation, PCR and NGS (**e**). Error bars indicate one s.d. based on measuring each of the five pools with a given expected read fraction.

To quantify the relative incorporation of strands into a DNA structure, one needs a metric that accounts for ligation and NGS sequence bias. There is some sequence bias inherent to NGS, as we obtain a distribution of read counts even for oligonucleotides mixed at equal stoichiometry (Supplementary Fig. 3). This is consistent with observations in other NGS library preparation methods that certain library members tend to be over- or under-represented based on their sequence^42^. Fortunately, the sequence bias of NGS tends to be consistent, due to sequence-specific increases in ligation or amplification efficiency based on our observations (Fig. 2) and those in the literature^42^,^43^. Thus, by analysing the data appropriately, one should be able to account for this bias.

To account for the sequence bias of NGS, we introduce a new metric for measuring the relative incorporation of strands into a DNA structure called structure-wide relative incorporability (SRI). SRI is calculated by dividing the read fraction of each strand in the product by the sum of its read fractions in the product and monomer bands. As a result, the SRI varies from 0 (no incorporation) to 1 (full incorporation, no reads in the monomer), with higher SRI values indicating better incorporation of a strand relative to the other strands in a structure. SRI makes two key assumptions: that the variation in stoichiometry in the input assembly reaction is lower than the variation in incorporation into structures, and that sum of the product and monomer bands is representative of the entire assembly reaction. It is important to emphasize that SRI measurements are relative and not absolute because they involve the ratios of read fractions between pairs of samples (that is, the product band and the monomer band), which are effectively ratios of ratios. Thus, SRI is not a linear metric for strand incorporation, and should not be treated as such. Furthermore, it should be emphasized that the SRI is not equivalent to the bulk yield of a structure, as it is a nonlinear metric. For further details on SRI calculation, see the Methods section.

### Validating BEARS

BEARS is a reproducible and quantitative method for studying structure assembly. We assembled a 2D DNA brick structure on two separate occasions and prepared sequencing libraries from these replicate assemblies on separate days. We calculated SRI maps for each of these replicate assemblies using BEARS (Fig. 2a). Also, we directly compare the SRI for each brick strand in replicate structure assemblies using a scatterplot (Fig. 2b). The SRI values for each brick strand are highly correlated between replicate assemblies (correlation coefficient=0.925). This suggests that there is relatively little variation introduced to the incorporation data due to structure assembly, gel purification, library preparation and Illumina sequencing. It is likely that these variables would influence the monomer and product bands equally; hence, by taking the ratio of these samples, we can control for these sources of variation.

In addition to reproducibility, we also tested the quantitative performance of Illumina sequencing by mixing pools of pre-extended oligonucleotides with varying stoichiometries (Fig. 2c). This allowed us to directly amplify and sequence the mixes without any ligation, thereby testing the effects of amplification and NGS on quantification. We counted the number of reads for each pool of oligonucleotides in each mix, and calculated the read fraction for each pool by dividing the read counts for each pool by the total number of reads in the mix. We then compared the observed read fraction for each pool with the expected read fraction based on the stoichiometry in the mix, which was set to 0%, 10%, 20%, 30% or 40% based on the pipetted volume (Fig. 2d, see the Methods section for details). We found that the expected and observed read fractions were highly correlated (*R*^2^=0.9995), with a relatively low amount of variation between mixes (error bars indicate one s.d.). On the basis of the sizes of the error bars, we can detect 1.5-fold changes in stoichiometry with high reliability. This demonstrates that the amplification and sequencing parts of BEARS are fairly sensitive.

Ligation bias does not substantially alter quantification by sequencing. To test the effect of ligation bias on the quantitative performance of BEARS, we mixed pools of non-extended oligos with varying stoichiometry, and prepared libraries for sequencing using BEARS (see the Methods section for details). This allowed us to test the combined effects of ligation, amplification and NGS on quantification. As in Fig. 2d, we calculated read fractions for each pool, and compared the observed read fraction for each pool with the expected read fraction based on the stoichiometry in the mix (Fig. 2e). Although the correlation is less strong (*R*^2^=0.9921) than in the pre-extended case and the error bars are larger, we can still detect twofold changes in oligo stoichiometry using BEARS. In addition, we directly measured the ligation efficiency for 10 different oligonucleotides and found that the efficiency ranges from about 20 to 70% (Supplementary Fig. 2c). This indicates that ligation bias does add some variation to the read fractions measured using BEARS. Despite this, the method remains sensitive to twofold changes in oligo stoichiometry.

### Using BEARS to analyse 2D DNA brick structures

**Figure 3:**
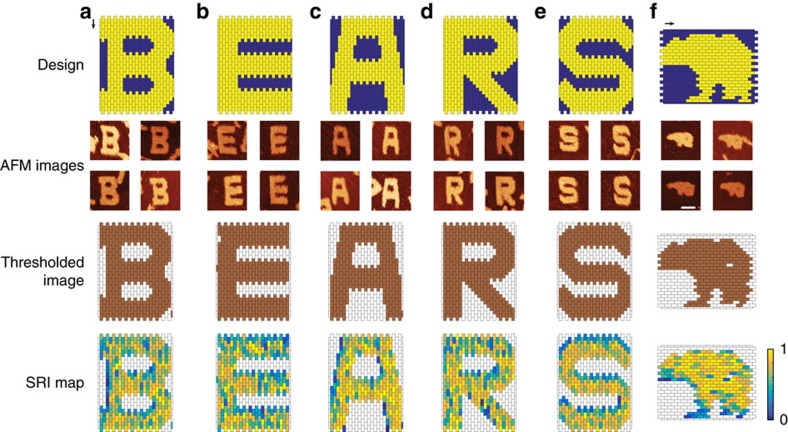
BEARS recapitulates the shape and SRI of bricks into 2D DNA brick structures. We tested six structures: the letter B (**a**), the letter E (**b**), the letter A (**c**), the letter R (**d**), the letter S (**e**) and a bear shape (**f**). In each panel, we show design schematics (top row), four representative AFM images of fully assembled structures (second row), thresholded images generated from SRI data (third row) and SRI data from BEARS (bottom row). Each box in the schematics and incorporation renderings indicates one brick. In the AFM images, the scale bars are 50 nm long. Arrows indicate the helical direction of each DNA brick in the five letters and the bear.

We can reconstruct structures from a 2D molecular canvas with high accuracy using BEARS. We assembled five arbitrarily chosen letters from a 2D molecular canvas^8^ (the canvas consists of ‘pixels' that are individual DNA bricks and can be modularly combined to form a variety of shapes) and one newly-designed bear shape using a subset of the DNA bricks from the same molecular canvas, and prepared sequencing libraries from both product and monomer bands using BEARS (Fig. 3). Each panel shows a design schematic, five representative AFM images of fully assembled structures, thresholded images based on Gaussian fitting of read fraction ratios and renderings of the SRI data (see the Methods section for details) for that structure generated using BEARS. When we computed histograms of the number of reads per brick strand for each structure design, we observed a bimodal lognormal distribution, with the rightmost peak corresponding to brick strands found in the structure and the left-most peak corresponding to reads from other brick strands in the molecular canvas. We used this distribution to set thresholds for rendering the SRI data for each structure. SRI values are displayed on a colour scale ranging from 0 (blue) to 1 (yellow), which are the maximum and minimum possible SRI values, respectively.

We observed good correspondence between the designs, AFM data and the thresholded sequencing data for all of the structures (Fig. 3a–f, first three rows). In a few cases, we obtained sequencing reads that did not correspond to brick strands found in a particular design. Conversely, we failed to detect fewer than 1% of brick strands in any given structure design. Reads corresponding to brick strands not found in a shape could come from contamination present in the strand mix, or could be introduced during sample processing. Brick strands within a shape that were not detected in either the monomer or product band by sequencing likely have poor ligation efficiency, and were more prevalent in samples with lower sequencing depth. For the bear design, there is a single missing brick strand in the hindquarters that is observed both in the AFM images and in the thresholded sequencing data (Fig. 3f). Together, these data indicate that BEARS can be used to reconstruct 2D DNA brick structures with single component resolution, which can sometimes be difficult to resolve with AFM.

SRI data from BEARS are recapitulated at the single structure level by AFM data. After analysing the SRI data (Fig. 3, fourth row), we noticed that certain structures had some areas with poor incorporation (blue), predominantly found near the edges of structures or in thinner features (for example, the legs of the bear in Fig. 3f). There is even a missing brick that is neither detected by BEARS or AFM analysis, suggesting that it was likely not present in the assembly reaction. We were able to find a number of partially assembled structures that were observed to be missing these poorly incorporated areas when imaged with the AFM (Supplementary Fig. 5). However, we did not observe a correlation between GC content or strand ΔG and SRI (Supplementary Fig. 6). Since our method involves gel-purifying assembled structures, we conclude that the SRI data represent an average of the class of structures present in the product band.

### Using BEARS to analyse 3D DNA brick structures

**Figure 4:**
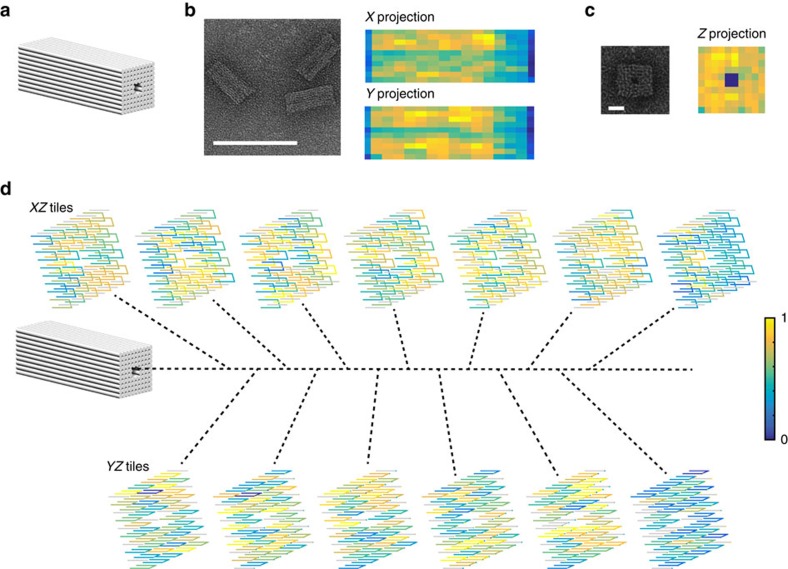
Extending BEARS to 3D DNA brick structures. We self-assembled a 3D DNA brick cuboid structure containing a hole along the long axis (**a**). We show TEM images of the structure and SRI data from BEARS are projected along each of the three major dimensions using MATLAB (**b**,**c**). Scale bar, 100 nm (**b**) and 10 nm (**c**). It is not possible to distinguish the *X* projections and *Y* projections using the TEM. We render incorporation data from BEARS in three dimensions for each layer of the structure using MATLAB (**d**). The location and order of each layer are indicated using the dotted lines. Spacing between adjacent helices is set to 2 nm, and each layer is ∼8.1 nm thick.

BEARS can be used to analyse 3D structures, including strands on the inside of the structures. Such analysis would be difficult to obtain using standard TEM methods. We assembled a 3D DNA brick cuboid with a tunnel along the long axis (Fig. 4a), imaged the product band using TEM and sequenced the monomer and product bands using BEARS (Fig. 4b,c). We calculated the SRI of each brick strand as in Fig. 2e and projected the incorporation data along each of the major axes of the structure (Fig. 4b,c). The tunnel in the middle of the structure is clearly visible in the rendering from the appropriate perspective (Fig. 4c), as well as a stripe of decreased intensity from left to right along the long axis in the other two projections (Fig. 4b). This could indicate directional bias in DNA brick incorporation. Furthermore, the spatial patterns of incorporation can be visualized by displaying slices through the long axis of the structure (Fig. 4d, see Supplementary Fig. 7 for a larger image). Such data indicate that there is some spatial clustering present in the SRI data. In particular, the right edge of the structure contains two layers with poor SRI (Fig. 4d, blue slices). As with the 2D DNA brick structures, we did not observe a correlation between the strand GC content or free energy and the SRI measurements (Supplementary Fig. 8). These data highlight the power of a label-free approach, as many brick strands in the interior of the structure may interfere with structure assembly when labelled.

### Extending BEARS to DNA origami structures

**Figure 5:**
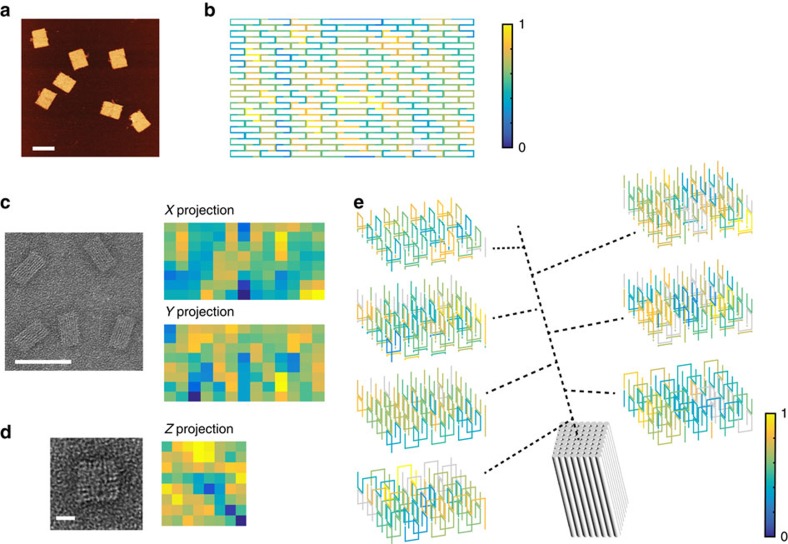
Extending BEARS to 2D and 3D DNA origami structures. We show AFM images and BEARS incorporation data for a 2D origami rectangle (**a**,**b**). Scale bar, 100 nm (**a**). We show TEM images and BEARS incorporation projections for a 3D origami cuboid (**c**,**d**). Scale bar, 50 nm (**c**); 10 nm (**d**). It is not possible to distinguish the *X* projections and *Y* projections using the TEM. We render SRI data from BEARS in three dimensions for slices through the 3D origami cuboid using MATLAB (**e**). The location and order of each slice is indicated using the dotted lines. Spacing between adjacent helices is set to 2 nm, and each slice is 5 nm thick.

BEARS can be used to calculate the SRI of staples in DNA origami structures. We assembled a 2D DNA origami rectangle (Fig. 5a,b) and a 3D DNA origami cuboid (Fig. 5c–e), imaged the product bands using AFM or TEM, respectively, and sequenced the monomer and product bands using BEARS. For a larger image of the slices of the 3D origami cuboid, see Supplementary Fig. 9. As with the DNA brick structures, we did not observe a correlation between the strand GC content or free energy and the SRI measurements (Supplementary Fig. 10). Also, we observe that there is less spatial correlation between SRI values in origami structures compared with DNA brick structures. Thus, we conclude that BEARS can be extended to measure the SRI of staples in DNA origami structures. Given this, we expect that BEARS will be extensible to a wide variety of DNA nanostructures with uniquely addressable components.

## Discussion

Here we have demonstrated a new method called BEARS for quantifying the component composition of DNA nanostructures. BEARS is high-throughput, label-free and generates data that correlate with AFM images of individual structures. We believe that BEARS is complementary to other structure characterization methods, such as AFM/TEM imaging and gel-based labelling. In particular, one can use BEARS to augment the resolution of a structure image, if one is not able to obtain component-level resolution by imaging. Alternatively, one can use BEARS to screen a new set of structure designs, and then image the designs with higher SRI values in areas of interest using AFM or TEM. In these ways, we envision that BEARS will help remove some of the existing bottlenecks in structure characterization, allowing one to design, build and test more structures than was previously possible.

One use case for BEARS is to determine the weak points of a structure design and improve them with a new design. This is particularly important for applications in which guest molecules are attached at specific points on a structure—these should be chosen to have the highest SRI or redesigned to optimize the yield. At present, the incorporation data represent an average of the class of structures purified from a product band on a gel. This provides a useful overview of which parts of a structure assemble well and which do not, but it does not provide sequencing data at the single structure level. One limitation of our method is that one needs a clear product band in order to ensure that the reads one generates come from properly assembled structures. However, this can likely be surpassed by fractionating an entire gel lane, or by using other methods for structure purification such as PEG precipitation or glycerol gradient centrifugation^44^,^45^. These types of improvements to BEARS will be driven by decreases in the cost of NGS, enabling one to sequence more samples in parallel, with higher depth per sample, thereby yielding better statistics about the self-assembly of populations of structures.

In addition to NGS, DNA synthesis is also experiencing a rapid decrease in price over time. One technology that will hopefully increase this trend is chip-based DNA synthesis, which allows for the production of tens of thousands of oligonucleotides at once^46^. These oligo libraries can then be used to assemble large libraries of regular-sized structures or larger structures themselves^47^. Larger structures can be difficult to characterize by imaging when the assembly yield is low, and it is easy to damage these structures during sample preparation and processing. However, even damaged structures could be quite informative, as portions of a structure that sustain more damage upon purification are likely to be weak points in a structure. Such structures are also of interest because they tend to assemble with lower yield, possibly suggesting that their assembly pathway(s) are very suboptimal, limiting the yield. Using BEARS, one could perhaps not only get component-level resolution renderings of these large structures but also improve the yields of the structures by redesigning the structures or changing the assembly conditions. Overall, a combination of single component resolution, high-throughput and built-in class averaging make BEARS a promising method for characterizing a wide variety of DNA nanostructures in the coming years.

## Methods

### Structure designs

The 2D DNA brick structures shown in Fig. 3 are derived from the R6 canvas described in ref. 8. The letters A, B, E, R and S were described in that work, whereas the bear structure was designed using a different subset of the R6 canvas. The 3D DNA brick structure shown in Fig. 4 has a 13 nt domain length. The 2D origami rectangle shown in Fig. 5 is based on a twist-corrected version^48^ of the original 2D origami rectangle^5^, but with a different scaffold sequence^32^. The 3D origami cuboid shown in Fig. 5 was designed using caDNAno. For staple or brick strand sequences and structure design schematics, see Supplementary Data 1.

### Structure assembly

The letters B, E, A, R and the bear shape were assembled in assembly buffer (5 mM Tris-HCl, 1 mM EDTA, adjusted to pH 8.0) supplemented with 25 mM MgCl_2_ using the following annealing protocol: 95 °C for 1 min, anneal from 90 to 60 °C at 5 min per degree, then from 60 to 25 °C at 25 min per degree, followed by a hold at 25 °C. For AFM imaging, the letter S was assembled in assembly buffer (5 mM Tris-HCl, 1 mM EDTA, adjusted to pH 8.0) supplemented with 25 mM MgCl_2_ using a modified annealing protocol: 95 °C for 1 min, anneal from 90 to 60 °C at 5 min per degree, then from 60 to 40 °C at 45 min per degree, followed by a hold at 25 °C.

For the 2D origami rectangle, scaffold (M13, purchased from Bayou Biolabs) and staples were mixed together at target concentrations of 10 and 100 nM, respectively, in TAE (40 mM Tris acetate, 1 mM EDTA) buffer with 12.5 mM magnesium acetate (TAE/Mg). For 2D origami folding, the mixtures were kept at 90 °C for 5 min and annealed from 90 to 60 °C over the course of 30 min, from 60 to 45 °C over the course of 90 min, and from 45 to 25 °C over the course of 20 min. For the 3D origami cuboid, scaffold (p7560 (ref. 13)) and staple strands were mixed at 10 and 100 nM, respectively, in assembly buffer (5 mM Tris-HCl, 1 mM EDTA, adjusted to pH 8.0) supplemented with 10 mM MgCl_2_ and annealed over 3 days using the following protocol: anneal from 80 to 60 °C at 2 min per degree, then from 60 to 25 °C at 2 h per degree. For the 3D DNA brick cuboid, brick strands were mixed together at 100 nM in assembly buffer (5 mM Tris-HCl, 1 mM EDTA, adjusted to pH 8.0) supplemented with 20 mM MgCl_2_ and annealed over 3 days using the following protocol: anneal from 80 to 60 °C at 2 min per degree, then from 60 to 25 °C at 2 h per degree.

### Gel electrophoresis

The 2D DNA brick shapes, 2D origami and 3D origami were analysed by electrophoresis in a native 1.5% agarose gel supplemented with 10 mM MgCl_2_. Electrophoresis was performed at 90 V for 2 h in an ice-water bath. Gels were pre-stained with 1 × Sybr Safe (Life Technologies). The 3D DNA brick cuboid was analysed by electrophoresis in a native 1% agarose gel supplemented with 10 mM MgCl_2_. Electrophoresis was performed at 80 V for 2 h in an ice-water bath. Gels were pre-stained with 1 × Sybr Safe (Life Technologies). Afterwards, gels were scanned with a Typhoon FLA 9000 (General Electric) using the SYBR Safe channel (excitation at 473 nm, emission ≥510 nm).

Gel bands were visualized using a Safe Imager 2.0 Blue-Light Transilluminator (Invitrogen) and excised from the gel using a fresh razor blade. The excised piece was then placed into a Freeze ‘N Squeeze column (Bio-Rad) and crushed using a plastic pestle (USA Scientific). For the 2D DNA brick shapes, 2D origami rectangle and the 3D origami cuboid, structures were eluted from the column by centrifugation at 400*g* for 3 min. For the 3D DNA brick cuboid, structures were eluted from the column by centrifugation at 1,200*g* for 3 min.

### Atomic force microscopy

Images of folded structures were obtained with a Veeco Multimode V atomic force microscope. C-type Bruker SNL-10 tips were used under tapping mode in fluid. Samples (25 μl) were deposited on the mica surface for 1 min. The mica surface was then rinsed five times with 0.5 × TE (5 mM Tris, 1 mM EDTA, adjusted to pH 8.0) supplemented with 25 mM MgCl_2_. For the 2D DNA brick shapes, samples were supplemented with 5 mM NiCl_2_ (final concentration) to aid in attachment to the mica surface before imaging. The 2D origami rectangle was imaged in 1 × TE (10 mM Tris, 1 mM EDTA, adjusted to pH 8.0) supplemented with 25 mM MgCl_2_.

### Transmission electron microscopy

A volume of 2.5 μl of each sample was deposited on to glow-discharged, carbon-coated EM grids for 2 min. The liquid was wicked off and 2.5 μl of stain (2% uranyl formate+25 mM NaOH) was added. The 3D DNA brick structure was stained for 30 s, and the 3D origami cuboid was stained for 45 s. After staining, excess liquid was wicked off. All samples were imaged using a JEOL JEM-1400 TEM operating at 80 kV.

### Oligonucleotide mixing experiments

For the experiments described in Fig. 2d, 30 oligonucleotides of length 80 nt were divided into five pools of six oligonucleotides each. These pools, 1–5, were mixed together with systematically varying stoichiometry as follows: Mix A 1:1:1:1:1, Mix B 1:2:3:4:0, Mix C 0:1:2:3:4, Mix D 4:0:1:2:3, Mix E 3:4:0:1:2 and Mix F 2:3:4:0:1. Thus, in each mix, each pool is present at 0, 10, 20, 30 or 40% of the total mix. The mixes A–F were amplified using two cycles of PCR and sequenced.

For the experiments described in Fig. 2e, 30 oligonucleotides of length 42 nt were divided into five pools of six oligonucleotides each. These pools, 1–5, were mixed together with systematically varying stoichiometry as follows: Mix A0 1:1:1:1:1, Mix B0 1:2:3:4:0, Mix C0 0:1:2:3:4, Mix D0 4:0:1:2:3, Mix E0 3:4:0:1:2 and Mix F0 2:3:4:0:1. Thus, in each mix, each pool is present at 0, 10, 20, 30 or 40% of the total mix. The mixes A–F were prepared for sequencing using the full BEARS protocol (see below).

Data were analysed by calculating the average number of reads per oligo in each pool, then dividing the read fractions in mixes B–F by the read fractions calculated from Mix A, which contains each oligo pool mixed with a 1:1:1:1:1 stoichiometry. The resulting normalized read fractions were compared with the expected read fractions based on the mix stoichiometries. The data shown in Fig. 2d,e are the mean normalized read fraction for each of the five pools at each expected read fraction, based on the normalized data from mixes B–F (see formula below: RF indicates read fraction and NRF indicates normalized read fraction). Error bars were calculated by taking the s.d. from the five pools with a given expected read fraction.


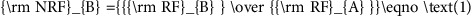


### Sequencing library preparation

Gel-purified monomer or product bands were concentrated using the Oligo Clean and Concentrator kit (Zymo Research), eluting with 6 μl of Milli-Q H_2_O. Samples were denatured by heating to 95 °C for 5 min. The 3 dC adaptor sequence (see Supplementary Table 1 for details) was then ligated to the 3′-ends of the staples/brick strands using T4 RNA ligase 1 (New England Biolabs). Each 10 μl ligation reaction contained 10 units of enzyme, 25% (w/v) PEG-8000, 1 × T4 RNA ligase buffer, 5 pmol of the 3 dC adaptor, 2–5 pmol of brick strands/staples and ATP at a final concentration of 1 mM. Ligation reactions were incubated overnight at room temperature, then heat-inactivated at 65 °C for 20 min.

After heat-inactivation, samples were analysed using denaturing polyacrylamide gel electrophoresis (Supplementary Fig. 2a). Samples were mixed 1:1 with 2 × RNA loading dye (New England Biolabs), denatured for 10 min at 70 °C and loaded onto a precast 10% TBE-urea gel. Electrophoresis was performed at 65 °C for 35–55 min at 180 volts using 0.5 × TBE as a running buffer. Gels were post-stained with 1 × Sybr Gold (Invitrogen) for 30 min in an orbital shaker. Ligation product bands were visualized using a Safe Imager 2.0 Blue-Light Transilluminator (Invitrogen), and excised from the gel using razor blades or 1.1 × 6.5 mm gel cutting tips (MidSci).

Gel slices were placed into dialysis tubes (Slide-A-Lyzer MINI Dialysis Device, 2K MWCO, 0.1 ml) and 50 μl of 0.5 × TBE was added to submerge the gel slices. Electroelution was performed at 90 V for 30 min in 0.5 × TBE, followed by reversing the leads and running for ∼30 s to prevent the DNA from being stuck to the surface of the dialysis tube. Electroeluates were then concentrated using the Oligo Clean and Concentrate kit (Zymo Research), eluting with 6 μl of Milli-Q H_2_O.

The gel-purified ligation product was phosphorylated using T4 polynucleotide kinase (New England Biolabs). Phosphorylation reactions were carried out in a 10 μl reaction volume containing 5–10 units of enzyme, 1 × T4 PNK buffer and 1 mM final concentration of ATP. Reactions were incubated for 30 min at 37 °C, then heat-inactivated at 65 °C for 20 min.

The 5 dC adaptor sequence (containing indexing barcodes, see Supplementary Table 1 for details) were then ligated to the 3′ ends of the staples/brick strands using T4 RNA ligase 1 (New England Biolabs). Each 20 μl ligation reaction contained 2 μl of enzyme at 10 U μl^−1^, 10 μl of 50% (w/v) PEG-8000 (final concentration: 25% (w/v)), 2 μl of 10 × T4 RNA ligase buffer, 5 pmol of the 3 dC (1 μl at 5 μM), 3 μl of phosphorylated ligation 1 product and 2 μl of 10 mM ATP (final concentration: 1 mM). Ligation reactions were incubated overnight at room temperature, then heat-inactivated at 65 °C for 20 min. We then purified the samples using the Oligo Clean and Concentrate kit (Zymo Research), eluting with 6–10 μl of Milli-Q H_2_O.

### Library amplification and quantification

Individual samples were amplified before pooling using Q5 polymerase (from 2 × master mix purchased from NEB) and previously validated Illumina qPCR primers at a final concentration of 300 nM. Between 2 and 10 μl of template was used in a 50 μl PCR reaction. Sequencing libraries were quantified using quantitative PCR. Q5 polymerase (NEB) was used according to the manufacturer's instructions. Previously validated Illumina qPCR primers^43^ were synthesized by IDT and used at a final concentration of 300 nM. Syto13 (Molecular Probes/Life Technologies) was used as a fluorescent indicator dye according to the manufacturer's instructions. DNA standards 1–6 from the Kapa NGS library quantification kit were used to make a standard curve for absolute concentration determination (sample qPCR data are shown in Supplementary Fig. 2b).

### Next-generation sequencing

Libraries were sequenced using an Illumina MiSeq machine according to the manufacturer's instructions using the MiSeq V2 paired end 50 kit (Illumina Inc., San Diego, CA). In some cases, we used a modified library denaturation and loading protocol optimized for lower-concentration libraries^43^.

### Data analysis

Sequence processing was done using custom MATLAB software. Fastq files from the MiSeq were parsed and partitioned based on the index reads. Sequence matching was done using regular expressions to query for an exact match to the first 20 bases of a brick strand or staple, allowing between 4 and 7 random nucleotides at the 5′-end of the read. These random nucleotides are part of the 5′-sequencing adaptor, and add sequence diversity to first few cycles of the sequencing (necessary for the machine to focus properly). Data were filtered by disregarding strands with fewer than 25 reads present in the product band, as there is not sufficient information to accurately quantify the incorporation for these strands. Read counts were normalized to the overall number of reads in a sample, resulting in the ‘read fraction' data for both the product and monomer bands.

After calculating the read fraction for each strand in a structure, we applied a threshold to determine which strands were actually present in a structure and which were not. Specifically, we took the ratio of the read fraction for each strand in the product band and the read fraction for each strand in the canvas mix. This resulted in a bimodal lognormal distribution for most shapes, since they contain a subset of all of the strands in the canvas. By taking the ratio, we control for how well each individual strand is ligated, amplified and sequenced. Thresholds were determined by fitting the sum of two Gaussian distributions to histograms (with 20 evenly spaced bins) of the log_2_ of the read fraction ratio (product/canvas) mentioned above using MATLAB. Specifically, threshold was taken as the minimum value of the sum of the Gaussian fits, rounded up to by the width of one bin from the histogram. Strands that passed the threshold were analysed further, as described below.

SRI values were calculated based on the ratio of the read fraction in the product band, divided by the sum of the read fractions in the monomer and product bands (see formula).


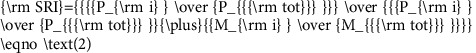


Where *P*_i_ and *M*_i_ are the read counts for oligonucleotide *i* in the product and monomer bands, respectively, and *P*_tot_ and *M*_tot_ are the total read counts in the product and monomer bands. We divide *P*_i_ and *M*_i_ by P_tot_ and *M*_tot_, respectively, because the total number of reads varies between samples; thus, the read count is inherently a relative measure rather than an absolute measure. This metric has two key assumptions. The first assumption is that the variation in the starting concentrations of the oligonucleotides used for self-assembly is lower than the variation expected to be found in the product band. Otherwise, the method will tend to measure variation in the starting stoichiometry, rather than in the relative incorporability of DNA oligonucleotides into the structures themselves. The second assumption is that the even distribution of oligonucleotide stoichiometry is not influenced by aggregation. If aggregation is strand-specific, then certain oligonucleotide might get sequestered preferentially in aggregates, thereby biasing the incorporation measurements for those oligonucleotides. So long as these two assumptions hold, the SRI is a good proxy for the relative incorporability of an oligonucleotide in a DNA nanostructure. We expect the variation in starting stoichiometry to be small (∼10%) based on spectrophotometric measurements of oligo concentrations. Also, we expect that aggregation will not be strand-specific, as it is likely mediated by nonspecific interactions between oligonucleotides or structures.

### Structure rendering

Coordinates of each strand were parsed from the caDNAno design files using custom MATLAB software, and are coloured based on the SRI of each brick strand or staple. Brick strands with fewer than 25 reads in the product band are coloured grey. Half-brick strands and edge protector brick strands are also coloured grey. Data are rendered using an inter-helix distance of 2 and 10.67 nm per helical turn, based on the square lattice model.

### Data availability

The data that support the findings of this study are available within this article and it Supplementary Information files and from the corresponding author upon reasonable request. NGS data are available at the ENA database, accession no.: PRJEB18731. Source code for NGS data analysis is available from the corresponding author upon reasonable request.

## Additional information

**How to cite this article:** Myhrvold, C. *et al*. Barcode extension for analysis and reconstruction of structures. *Nat. Commun.* **8,** 14698 doi: 10.1038/ncomms14698 (2017).

**Publisher's note:** Springer Nature remains neutral with regard to jurisdictional claims in published maps and institutional affiliations.

## Supplementary Material

Supplementary Information Supplementary Figures and Supplementary Table.

Supplementary Data 1 non-extended oligos and pre-extended oligos.
