# Supplementary material for: Barcode extension for analysis and reconstruction of structures
Source: Nat Commun. 2017 Mar 13;8:14698. doi: 10.1038/ncomms14698 (PMC5355802; doi:10.1038/ncomms14698)
Supplement: Supplementary Information — Supplementary Figures and Supplementary Table. [file ncomms14698-s1.pdf]

Supplementary Table 1. Adapter and barcode sequences for BEARS.

**Name**	**Sequence**
phos_r2_ddC	/5Phos/AGATCGGAAGAGCACACGTCTGAACTCCAGTCACATCTCGTATGCCGTCTTCTGCTTG/3ddC/
r1_bc_full_1	AATGATACGGCGACCACCGAGATCTACACAACGATGGACACTCTTTCCCTACACGACGCTCTTCCGATCTNNNN
r1_bc_full_2	AATGATACGGCGACCACCGAGATCTACACACCTTGGAACACTCTTTCCCTACACGACGCTCTTCCGATCTNNNNN
r1_bc_full_3	AATGATACGGCGACCACCGAGATCTACACACGTTCCTACACTCTTTCCCTACACGACGCTCTTCCGATCTNNNNNN
r1_bc_full_4	AATGATACGGCGACCACCGAGATCTACACAGACTGTCACACTCTTTCCCTACACGACGCTCTTCCGATCTNNNNNNN
r1_bc_full_5	AATGATACGGCGACCACCGAGATCTACACAGGTGTTCACACTCTTTCCCTACACGACGCTCTTCCGATCTNNNN
r1_bc_full_6	AATGATACGGCGACCACCGAGATCTACACATCGCCTTACACTCTTTCCCTACACGACGCTCTTCCGATCTNNNNN
r1_bc_full_7	AATGATACGGCGACCACCGAGATCTACACCAACCTTCACACTCTTTCCCTACACGACGCTCTTCCGATCTNNNNNN
r1_bc_full_8	AATGATACGGCGACCACCGAGATCTACACCAGAGACAACACTCTTTCCCTACACGACGCTCTTCCGATCTNNNNNNN
r1_bc_full_9	AATGATACGGCGACCACCGAGATCTACACCATGCATGACACTCTTTCCCTACACGACGCTCTTCCGATCTNNNN
r1_bc_full_10	AATGATACGGCGACCACCGAGATCTACACCGAATACCACACTCTTTCCCTACACGACGCTCTTCCGATCTNNNNN
r1_bc_full_11	AATGATACGGCGACCACCGAGATCTACACCTAGAGGAACACTCTTTCCCTACACGACGCTCTTCCGATCTNNNNNN
r1_bc_full_12	AATGATACGGCGACCACCGAGATCTACACCTTCAGCTACACTCTTTCCCTACACGACGCTCTTCCGATCTNNNNNNN
r1_bc_full_13	AATGATACGGCGACCACCGAGATCTACACGACTCAGTACACTCTTTCCCTACACGACGCTCTTCCGATCTNNNN
r1_bc_full_14	AATGATACGGCGACCACCGAGATCTACACGCAACCTAACACTCTTTCCCTACACGACGCTCTTCCGATCTNNNNN
r1_bc_full_15	AATGATACGGCGACCACCGAGATCTACACGCTAGCATACACTCTTTCCCTACACGACGCTCTTCCGATCTNNNNNN
r1_bc_full_16	AATGATACGGCGACCACCGAGATCTACACGGCGAATAACACTCTTTCCCTACACGACGCTCTTCCGATCTNNNNNNN
r1_bc_full_17	AATGATACGGCGACCACCGAGATCTACACGTACGAAGACACTCTTTCCCTACACGACGCTCTTCCGATCTNNNN
r1_bc_full_18	AATGATACGGCGACCACCGAGATCTACACGTGAGTGAACACTCTTTCCCTACACGACGCTCTTCCGATCTNNNNN
r1_bc_full_19	AATGATACGGCGACCACCGAGATCTACACGTTGGATCACACTCTTTCCCTACACGACGCTCTTCCGATCTNNNNNN
r1_bc_full_20	AATGATACGGCGACCACCGAGATCTACACTAGGTAGGACACTCTTTCCCTACACGACGCTCTTCCGATCTNNNNNNN
r1_bc_full_21	AATGATACGGCGACCACCGAGATCTACACTCCTCTTCACACTCTTTCCCTACACGACGCTCTTCCGATCTNNNN
r1_bc_full_22	AATGATACGGCGACCACCGAGATCTACACTCTCGTCAACACTCTTTCCCTACACGACGCTCTTCCGATCTNNNNN
r1_bc_full_23	AATGATACGGCGACCACCGAGATCTACACTGAGGTCTACACTCTTTCCCTACACGACGCTCTTCCGATCTNNNNNN
r1_bc_full_24	AATGATACGGCGACCACCGAGATCTACACTGTGTGACACACTCTTTCCCTACACGACGCTCTTCCGATCTNNNNNNN

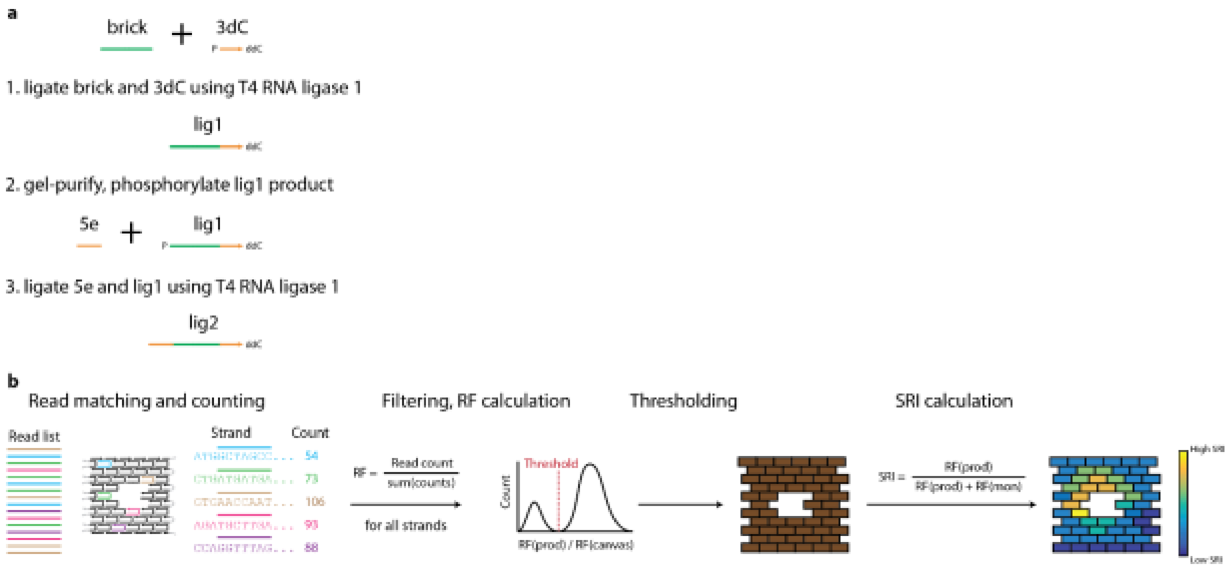

Supplementary Figure 1. Detailed schematic of BEARS ligations and data analysis. We show a schematic (a) of the first ligation, between a brick and the 3’ dideoxyC-protected adapter (3dC), resulting in the lig1 product. The lig1 product is then gel-purified, phosphorylated, and ligated to the barcoded 5e adapter, resulting in the lig2 product. We also show details of the data analysis pipeline (b).

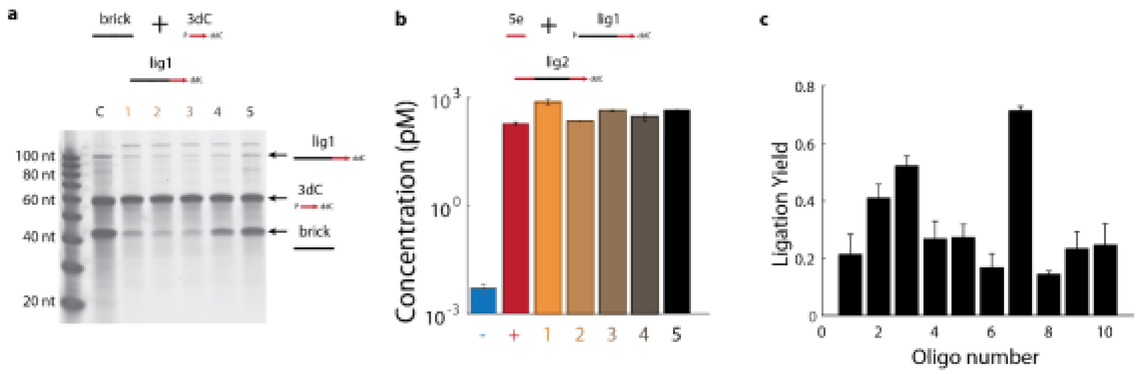

Supplementary Figure 2. Denaturing gel and qPCR analysis of BEARS ligations. We show a denaturing PAGE gel of the first ligation reaction (a), in which 42-nt bricks are ligated to ~60 nt 3dC adapters, resulting in the lig1 product, shown near the top. The numbers 1-5 indicate different samples that were analyzed using BEARS. The second ligation product was quantified using qPCR. We typically observed sample concentrations between 100 and 1000 pM (orange, brown, grey, black), at similar levels to the positive controls (red), and substantially higher than the negative controls (blue). We tested the ligation efficiency of 10 different oligonucleotide sequences (c). Error bars indicate 1 standard deviation based on n=3 experiments.

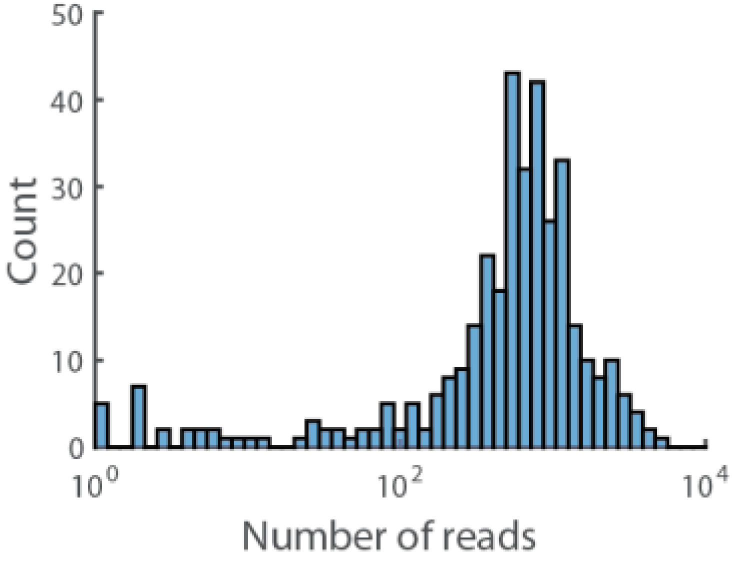

Supplementary Figure 3. We show a sample read count histogram for the 2D DNA brick molecular canvas. We observe a lognormal distribution for the majority of bricks, with a few outliers that are processed or sequenced poorly.

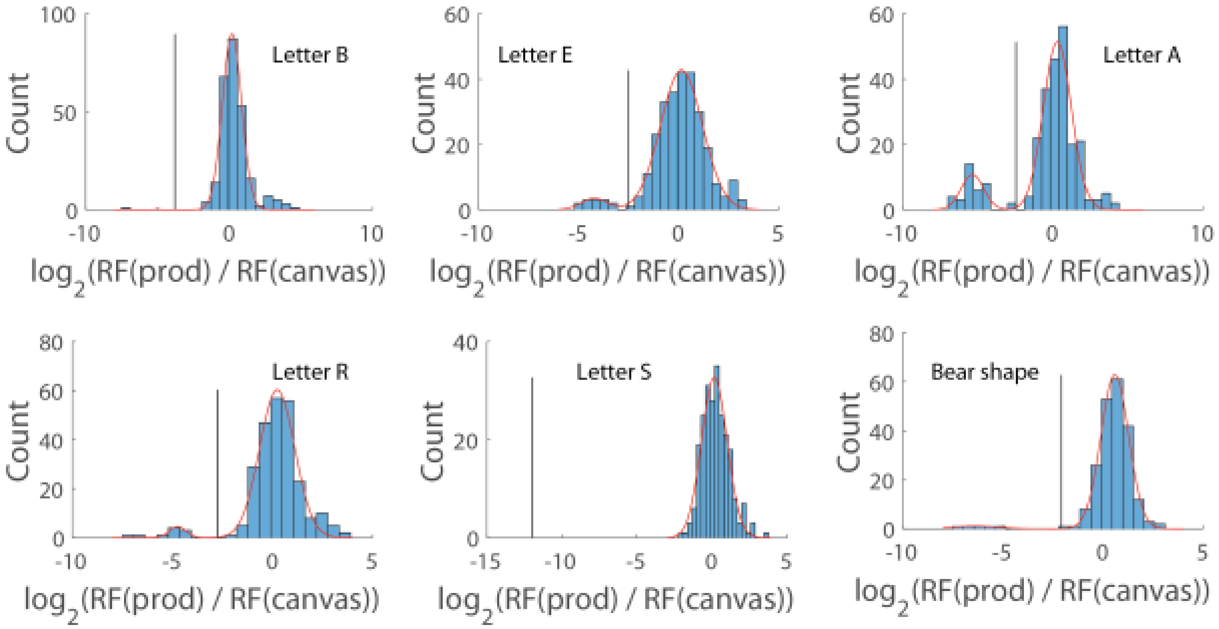

Supplementary Figure 4. Read fraction distributions of the 2D DNA brick structures. We show histograms of the $\log_2$ of the read fraction ratios for each of the 2D shapes from Fig. 3. Gaussian curve fits are indicated in red, and the threshold is shown using a black line.

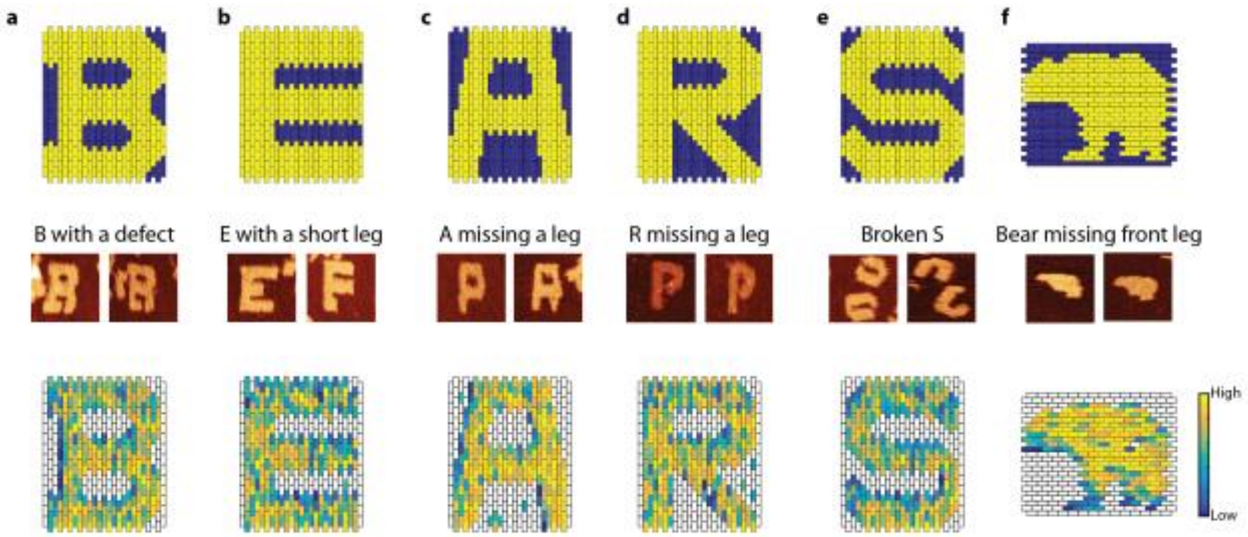

Supplementary Figure 5. Low SRI values correlate with structure defects. We show structure schematics and SRI data from Figure 3, along with representative AFM images of defective structures. The break points and edges of these structures correspond to areas of low SRI.

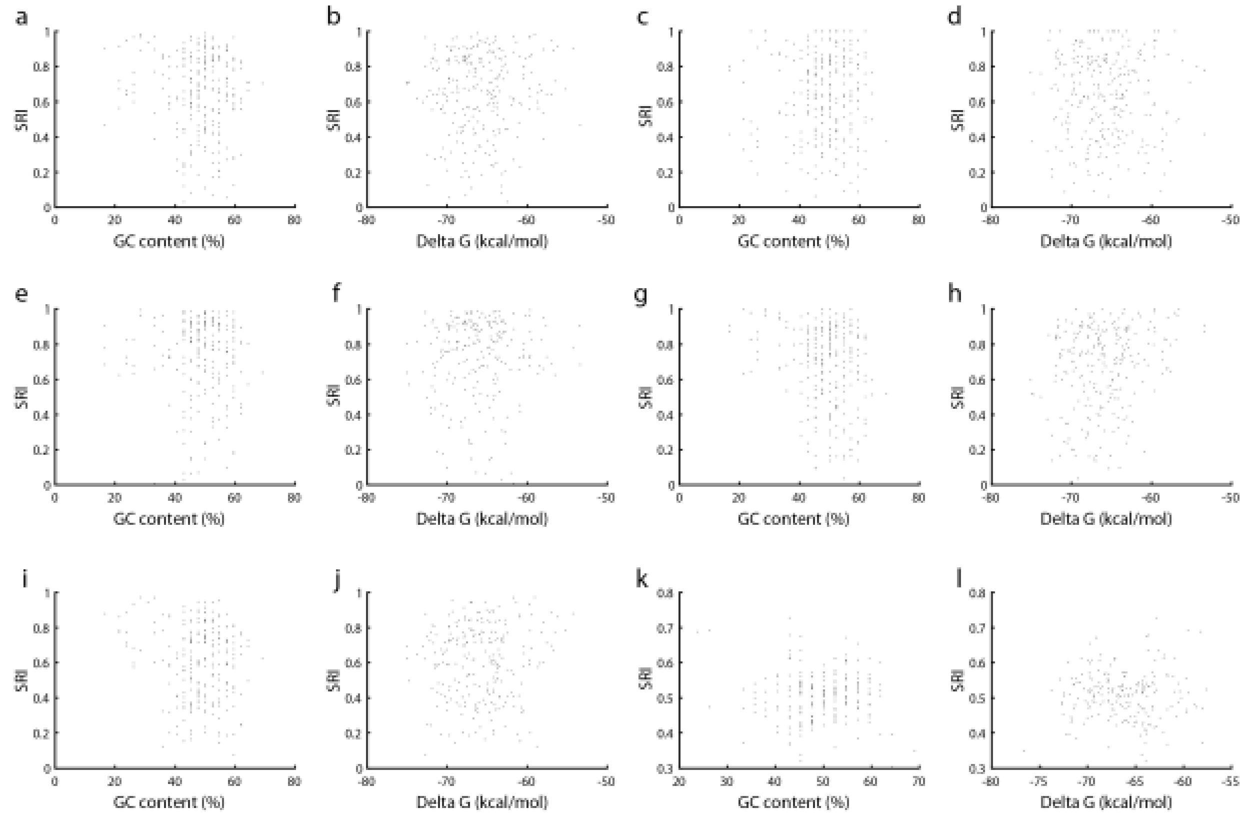

Supplementary Figure 6. GC content and free energy do not correlate with SRI for the DNA brick shapes. We show scatterplots of GC content vs. SRI and ΔG vs. SRI for the letter B (a-b), letter E (c-d), letter A (e-f), letter R (g-h), letter S (i-j), and bear shape (k-l).

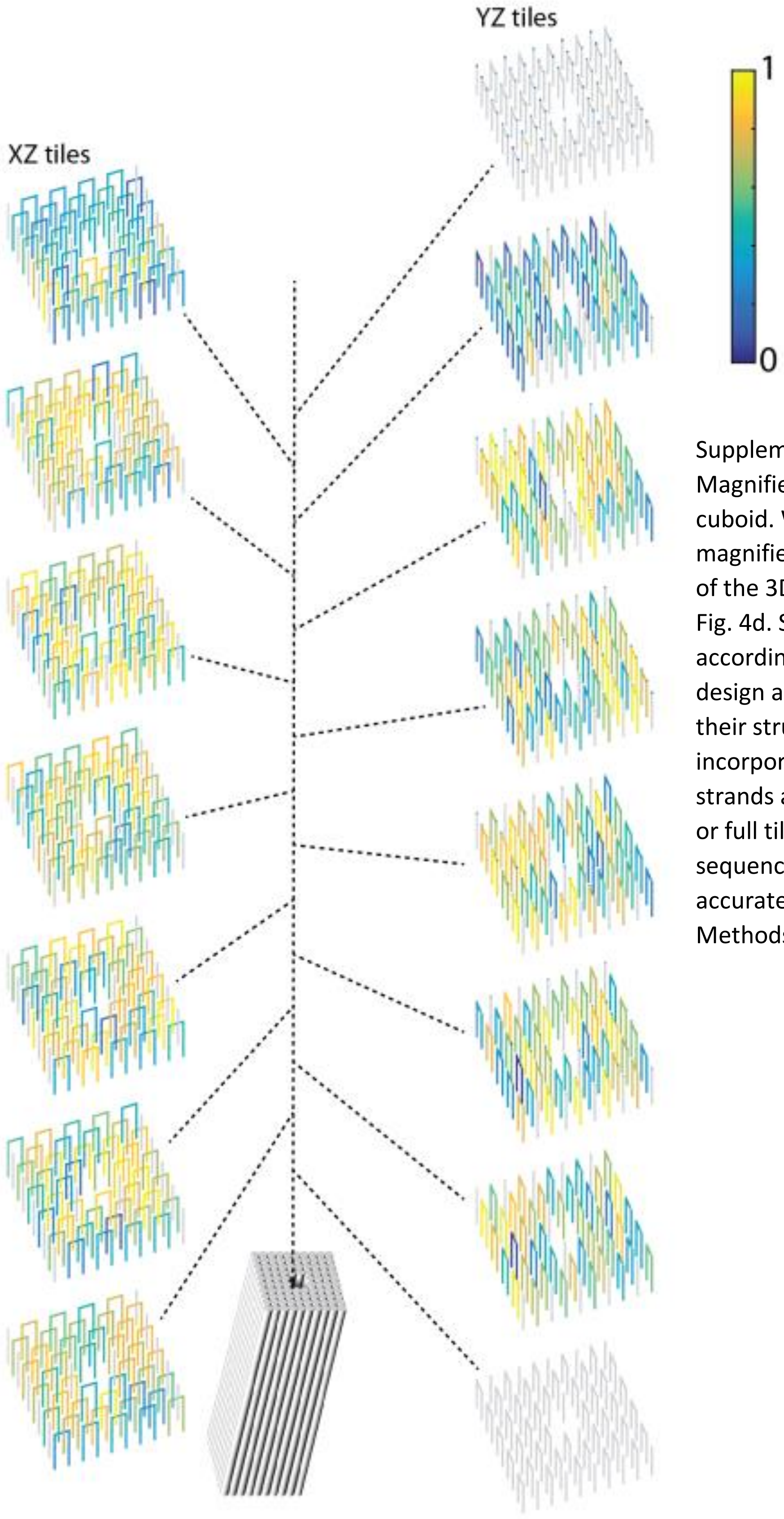

Supplementary Figure 7. Magnified view of 3D SST cuboid. We show a magnified view of the slices of the 3D SST cuboid from Fig. 4d. Strands are rendered according to the caDNAno design and colored based on their structurwise relative incorporability (SRI). Gray strands are either half-tiles or full tiles with too few sequencing reads for accurate quantification (see Methods for details).

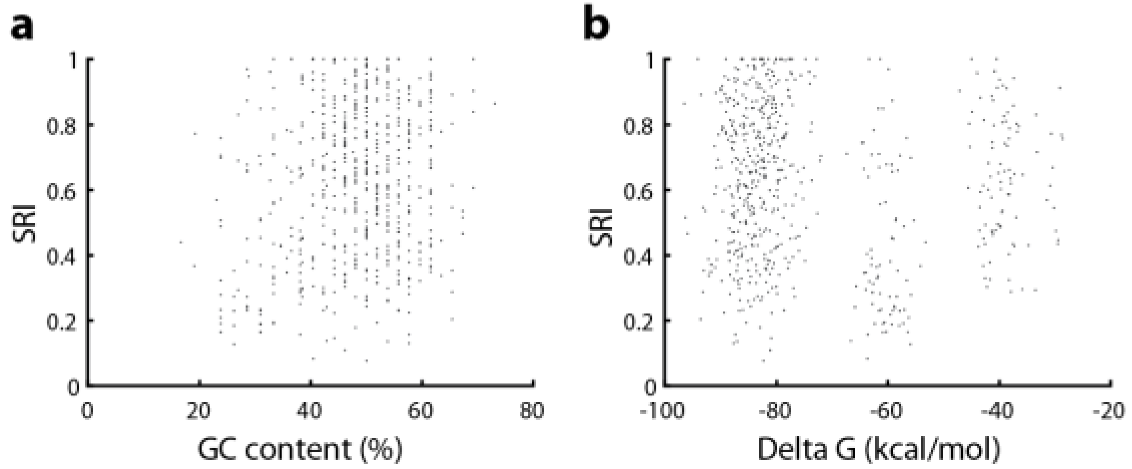

Supplementary Figure 8. GC content and free energy do not correlate with SRI for the 3D DNA brick cuboid. We show scatterplots of GC content vs. SRI and ΔG vs. SRI for the 3D DNA brick cuboid (a-b).

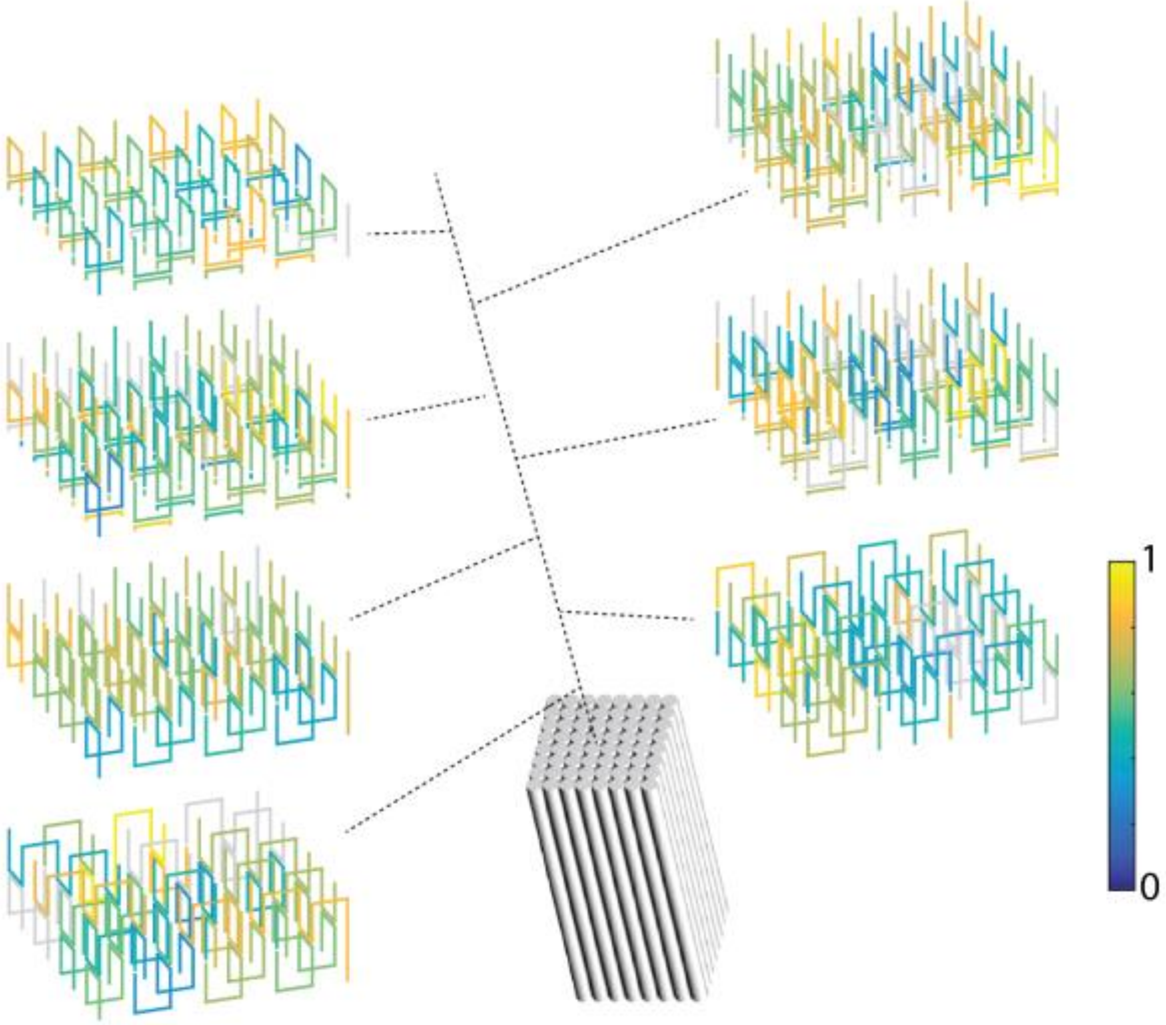

Supplementary Figure 9. Magnified view of 3D origami cuboid. We show a magnified view of slices of the 3D origami cuboid from Fig. 5e. Staples are rendered according to the caDNAno design and colored based on their structure-wise relative incorporability (SRI). Gray strands did not have enough reads for accurate quantification (see Methods for details).

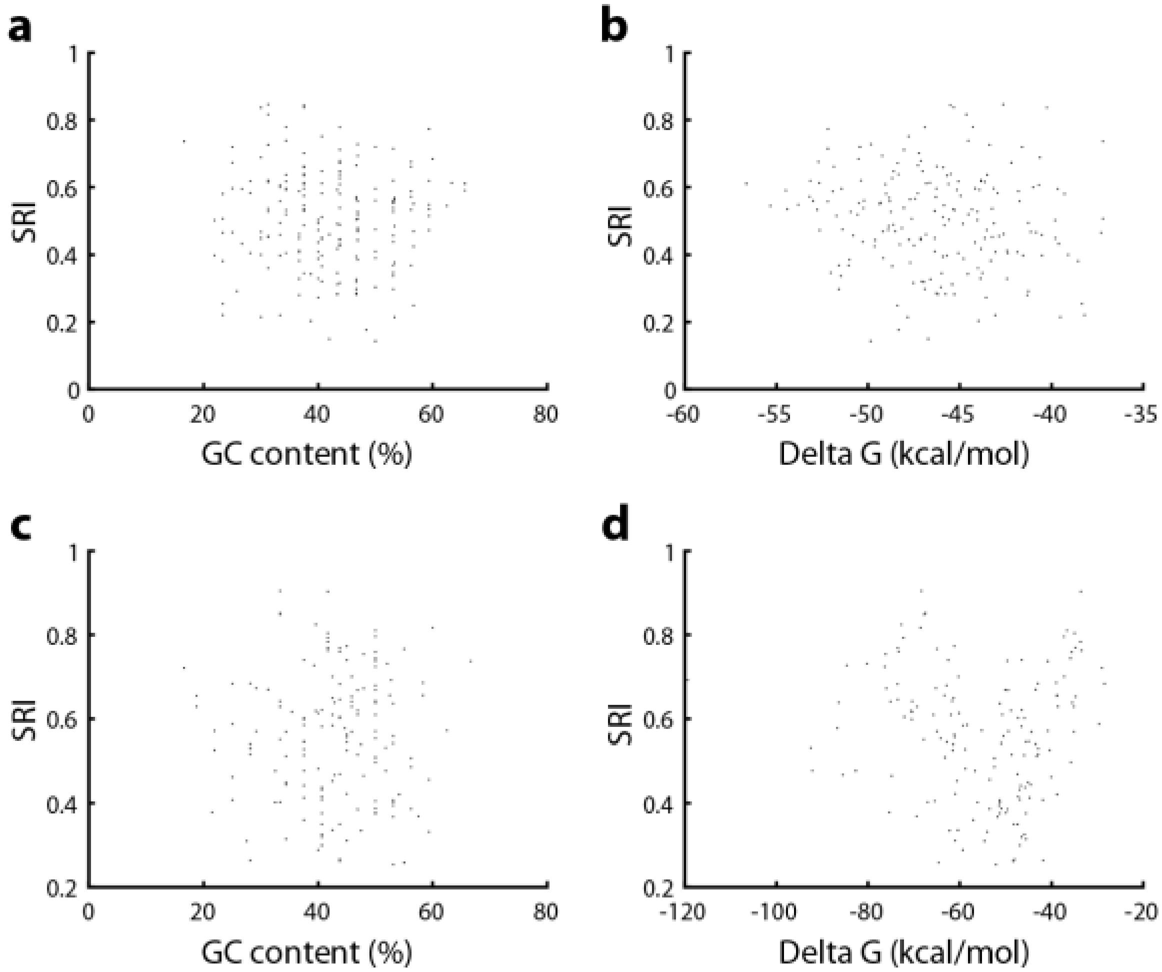

Supplementary Figure 10. Staple GC content and free energy do not correlate with SRI. We show scatterplots of GC content vs. SRI and ΔG vs. SRI for the 2D rectangular DNA origami (a-b), and the 3D DNA origami cuboid (c-d).
